# Dietary tea tree (*Melaleucae Aetheroleum*) oil fortifies growth, biochemical, immune-antioxidant trait, gene function, tissue reaction, and *Aeromonas sobria* resistance in Nile tilapia (*Oreochromis niloticus*)

**DOI:** 10.1186/s12917-024-04369-1

**Published:** 2025-01-02

**Authors:** Mohamed Shaalan, Heba H. Mahboub, Ahmed Hosny Abdelgawad, Abdelwahab A. Abdelwarith, Elsayed M. Younis, Ahmed A. Elnegiry, Asmaa W. Basher, Walaa El-Houseiny, Sherif M. Shawky, Sahar H. Orabi, Simon J. Davies, Yasmina K. Mahmoud

**Affiliations:** 1 Department of Pathology, Faculty of Veterinary Medicine, Cairo University, Giza, 12211 Egypt; 2 Polymer Institute, Slovak Academy of Sciences, Dúbravská Cesta 9, 845 41 Bratislava, Slovakia; 3 Department of Aquatic Animal Medicine, Faculty of Veterinary Medicine, Zagazig University, PO Box 44511, Zagazig, Egypt; 4 Department of Microbiology and Immunology, Faculty of Veterinary Medicine, Aswan University, 81528, Aswan, Egypt; 5 Department of Zoology, College of Science, King Saud University, PO Box 2455, Riyadh, 11451 Saudi Arabia; 6 Department of Cytology and Histology, Faculty of Veterinary Medicine, Aswan University, 81528, Aswan, Egypt; 7 Department of Pharmacology, Faculty of Veterinary Medicine, South Valley University, Qena, 83523 Egypt; 8 Department of Physiology, Faculty of Veterinary Medicine, University of Sadat City, Sadat City, 32897 Menofia Egypt; 9 Department of Biochemistry and Chemistry of Nutrition, Faculty of Veterinary Medicine, University of Sadat City, Sadat City, 32897 Menofia Egypt; 10 Aquaculture Nutrition Research Unit ANRU, Ryan Institute, College of Science and Engineering, Carna Research Station, University of Galway, Galway, Ireland; 11 Department of Biochemistry and Molecular Biology, Faculty of Veterinary Medicine, Suez Canal University, Ismailia, Egypt

**Keywords:** Growth trait, Histo-biochemical indices, *Aeromonas sobria*, Nile tilapia, *Melaleucae Aetheroleum* oil

## Abstract

The current study had aimed to assess the long-term dietary supplementation with *Melaleucae aetheroleum*, tea tree essential oil (TTO). The impact on growth performance, biochemical indices, immune function, oxidant/antioxidant activity, gene expression, histopathology, and resistance against *Aeromonas sobria* in Nile tilapia (*Oreochromis niloticus*) was investigated. Four groups (with five replicates; G1 (control group, G2, G3, and G4) of Nile tilapia received diets enriched with TTO (doses of 0.0, 0.5, 1.0, and 2.0 mL/kg diet) for 60 days, then fish were challenged by *A. sobria*. Outcomes indicated an extensive elevation in growth metrics (final body weight, weight gain, SGR, feed intake and fish body protein). Similarly, the total blood protein, albumin, total globulin levels, Serum complement-3 levels, lysozyme activity, immunoglobulin M (IgM), nitric oxide, and phagocytic activity were significantly enhanced in all treatments, notably in the 2.0 mL TTO/kg fed groups compared to the control. Lower levels of urea, creatinine, AST, ALP, ALT, cortisol, glucose, triglycerides, cholesterol and body crude lipids were observed in the fish that were fed a 2.0 mL TTO/kg diet. Supplementing TTO at 2.0-mL /kg diet revealed the best results for elevating CAT, SOD, and GSH activities plus declining MDA value in hepatic homogenate. Additionally, dietary 2.0-mL TTO/kg showed the best outcomes for the intestinal morphometry plus maintaining the histological picture in spleen and liver. Concurrently, fish that were fed a 2.0 mL TTO/kg diet exhibited a substantial upregulation of *TNF-α*,* IL-1β*,* IL-10*,* TFG-β*,* IFN-γ* and *BCL-2* genes in the liver, while, *caspase-3*, and *BAX* were downregulated. Furthermore, TTO-enriched diets enhanced the relative percentage survival post-*A. sobria* challenge plus enhanced the clinical picture in a dose-dependent manner. Taken together, the findings revealed that long-term exposure to dietary TTO fortified the physiological performance, oxidant/anti-oxidant stability, immune function, gene expression, histological picture, and resistance of Nile tilapia against *A. sobria*.

## Introduction

Aquaculture has an essential role in offering exceptionally nutritious diet which is rich in minerals and vitamins [21]. *Oreochromis niloticus* is eminent as an omnivore fish belongs to the *Cichlidae* family. It is extensively cultivated worldwide for its higher nutritional and economic values [5]. In Egypt, it is the main cultured fish species which is distinguished by higher growth rate and potent resistance to environmental conditions [41].

There is a contradiction for using dietary chemical complements in aquaculture sector for combating bacterial diseases. As a result, the exploration of alternative herbal therapies especially essential oils is crucial for overcoming the huge costs, environmental, and toxicological impacts [54]. Among them, tea tree essential oil (TTO) is acquired from plant *Melaleuca alternifolia* which is characterized by different antibacterial, antioxidant, and therapeutic characteristics [27, 22, 30]. TTO is distinguished by potent antioxidant activity because of its antioxidant component; 4-terpineol [16]) besides it can prevent the production of superoxide in human monocytes and improve the activity of antioxidant enzymes in the piglets’ serum [56, 51]. Moreover, it has an essential role in metabolism of lipids, and accordingly lessens inflammatory reaction [53]. TTO has been tested as efficient antimicrobial agent to conquer the growth of various bacterial pathogens [23, 55].

In aquaculture practice, tea tree oil-dietary supplement has proved efficacy in enhancing the height of the intestinal villus of Nile tilapia [48]. Also, it boosts growth performance, non-specific immune response, and antioxidant activity of *Macrobrachium rosenbergii* [25]. In histological level, TTO can minimize gill histo-pathological injury, adjust activities of purinergic enzyme and develop the immune function in *Rhamdia quelen* diseased in *A. hydrophila* [12, 42].

Based on this instance, the present attempt was set up to assess the dietary supplementing impact of some concentrations of the TTO on growth efficacy, antioxidant activity, hepato-renal function, stress condition, protein and lipid profile, gene expression, and histopathological architecture in Nile tilapia. Besides, *Aeromonas sobria* resistance was evaluated after TTO dietary intervention.

## Materials and methods

### Recruiting and adjusting fish

A total of 300 *O. niloticus* specimens were acquired from the Abbassa fish hatchery located in Sharkia Province, Egypt, exhibiting apparent signs of well-being. Fish were transported alive using plastic waterproof bags, one-quarter of the bag was filled with water. The rest of the bag’s capacity was rich in oxygen which inserted into a plastic bag after addition of water, then sealed air-tight [29]. The fish had a mean initial body weight of 16.33 ± 0.76 g before the study began. Before the trial, the fish were acclimated for 15 days and then given a basic meal without any additional feed additives. Subsequently, the fish were placed randomly in glass aquaria with dimensions of 80 × 40 × 30 cm and filled with 70 L of tap water that had been treated to remove chlorine. The water was constantly aerated using an air stone connected to a central air compressor. The water parameters were continuously measured over the entire duration of the experiment. According to Eaton et al. [17], the values measured in the lab for fish growth were dissolved oxygen at 7.0 ± 0.5 mg/L, ammonia at 0.02 ± 0.004 mg/L, and nitrite at 0.017 ± 0.003 mg/L. A constant temperature of 26 ± 0.5 °C was maintained for the water. The lighting and dark cycles were regulated to last 12 h each.

### Preparing a diet

The control diet, which had 30% crude protein (CP), is detailed in Table 1 along with its precise chemical composition and components. The basal diet was supplemented with the tested TTO at four different levels (0, 0.5, 1.0, and 2.0 mL/kg diet), and the components were combined together to make a paste. The paste was ground into 1.5 mm pellets using a meat mincer. After 24 h of drying at room temperature, the pellets were put in the fridge at 4 °C until needed. Once the acclimatization period was over, 300 fish were evenly distributed into 4 groups of 75 fish each. Each group was then divided into 5 replicates of 15 fish. Three times daily, the fish in each tank were fed a diet equal to 3% of the total biomass. The metabolic wastes were removed by siphoning and each tank noted a daily water exchange of approximately 35%. Plus, the fish’s weight dictated a change in the feed every two weeks. The study lasted for sixty-days.

**Table 1:** Ingredients and proximate chemical analysis of the experimental diets (g/kg)

Ingredients	
Fish meal, 60%	100
Ground corn	240
Soybean meal 44%	420
Corn oil	15
Wheat bran	150
Fish oil	15
starch	50
Vitamin premix^a^	5
Mineral premix^b^	5
Total (g)	1000
**Chemical analysis**	
Crude protein (*N* ×6.25)	29.90
Crude lipids	9.90
Crude fiber	5.50
Ash	7.20
Nitrogen free extract (%)^c^	47.5
Gross energy (kcal/kg)^d^	4577.15

^a^Vitamin premix (per kg of premix): vitamin A,8,000,000 IU; vitamin E, 7000 mg; vitamin D_3_, 2,000,000 IU; vitamin K_3_,1500 mg; biotin, 50 mg; folic acid, 700 mg; nicotinic, 20,000 mg; pantothenic acid,7000 mg; vitamin B_1_, 700 mg; vitamin B_2_, 3500 mg; vitamin B_6_, 1000 mg; vitamin B_12_, 7 mg

^b^Mineral premix (per kg of premix): zinc sulfate, 4.0 g; iron sulfate, 20 g; manganese sulfate,5.3 g; copper sulfate, 2.7 g; calcium iodine, 0.34 g; sodium selenite, 70 mg; cobalt sulfate, 70 mg, and CaHPO_4_·2H_2_O up to 1 kg

^c^Calculated by difference (100 –protein% + lipids%+ ash% + crude fiber %)

^d^Gross energy (GE) was calculated as 5.65, 9.45 and 4.11 kcal/g for protein, lipid and NFE, respectively (NRC, 1993)

### Performance indicators for growth

By measuring the fish’s final weight (FW) and calculating their weight gain (WG), specific growth rate (SGR), feed intake (FI), and feed conversion ratio (FCR), productive performance of the fishes was assessed at the end of the feeding experiment (60 days) using the following formulas:

Specific growth rate (SGR) = 100 (Lin Wtf – Lin Wti) / days

where; Wtf = Weight of fish after 60 days (final) Wti = Weight of fish at beginning of experiment (initial)

Weight gain (WG) = Wtf (Final weight of fish) – Wti (Initial weight of fish)

Feed intake (FI) = feed consumed/Number of survival fish.

Feed conversion rate (FCR) = Total feed consumed by fish (g) / Weight gain by fish (g)

### Complete body chemical analysis

To ascertain the chemical components of the flesh, including crude lipid, moisture, crude protein, and ash, eight fish were randomly selected from each treatment following the feeding experiment, placed in plastic containers, and stored at -20 °C, as per AOAC [8]. The moisture content was determined after the dried weight was maintained at 105 °C through a drying process. The quantity of crude protein was ascertained by employing micro-Kjeldahl equipment. The total lipid concentration was calculated using the Soxhlet apparatus after a 16-hour petroleum ether extraction. The samples were subjected to combusting in a muffle furnace at a temperature of 550 °C for 6 h. Consequently, the quantity of ash present was quantified by utilizing the weight reduction.

### Sampling

Bio samples from the fish were taken after the feeding trial. To collect blood and lessen handling stress, the fish were sedated with tricaine methanesulfonate (MS-222, Argent Chemical Labs, Redmond, Washington, USA). To conduct the hematobiochemical tests, test tubes were coated with sterile EDTA, and ten fish were used, two from each replication, to collect whole blood from their caudal vasculature. Extra blood samples were collected (15 total), allowed to cool to room temperature, and subsequently spun at 3000 × g for 15 min to separate the serum, all without the use of an anticoagulant. All sera were frozen at -20 °C before being used in the hematobiochemical tests. Samples of the fish’s liver were removed aseptically after blood was drawn to assess oxidative stress evidence. Hepatic tissues were cleansed with cold, sterile saline, dried with filter paper, and kept on ice-cold plates. They were then frozen at -20 °C. After adding 100 mg of each tissue, a tube holding 1 ml of a buffer (10 mM phosphate/20 mM tris-pH 7.0) was filled. The mixture was then homogenized for 5 min at 4 °C at a speed of 6000 × g. The supernatants were combined and kept at -80 °C until needed after centrifugation. Liver, spleen, and mid-intestinal tissues from five fish per treatment were removed and stored in 10% neutral buffered formalin for histological analyses. Furthermore, a sterile dissection was conducted on ten fish from each group, and liver samples were collected after 60 days of feeding. The preceding samples were preserved for real-time PCR by placing them in an RNA later solution and storing them at a temperature of -20 °C.

### Analysis of biochemical markers in the serum

Colorimetrically measuring blood protein (total protein and albumin) yielded globulin levels by subtracting albumin from total protein. ALT, AST, and ALP were measured using commercial kits (Assay Kit, 384 well, Colorimetric/Fluorometric, ABACM241035), per Wilkinson et al. [50]. The levels of urea and creatinine were assessed using the protocol developed by Ajeniyi et al. [3]. Plasma triglyceride and cholesterol concentrations were determined using colorimetric and photometric enzymatic methods, respectively. Spectrum Bioscience (Egyptian Company for Biotechnology, Cairo, Egypt) provided the colorimetric diagnostic kits used to determine serum glucose and cortisol levels, following the procedures described by Trinder [47] and Tunn et al. [46].

### An evaluation of the immune system’s blood components

The levels of lysozyme activity were measured using turbidometric assays, as stated by Ellis [18]. We used ELISA kits from MyBioSource in San Diego, USA to measure the concentrations of Serum immunoglobulin M (IgM), nitric oxide (NO), and complement 3 (C3), following the instructions given by the manufacturer. Phagocytic activity was determined using Sakai et al. [39] technique. Anticoagulant-treated blood was mixed (1:1) with *Staphylococcus albus* (1.0 × 105 cells/ml) in PBS (PH 7.2) for 30 min at 37 oC. A drop of the mixture was flattened on a microscope slide. The cells were fixed in methanol for 30 min after drying. Three distilled water washes followed by 1–2 min of Levowitz-Weber staining. The data were analyzed with an oil immersion light microscope. Phagocytic cells graded bacteria consumption. A formula was used to measure phagocytosis activity: Number of phagocytosing cells/total cells x 100.

### Evaluation of the antioxidant and oxidative capacities of the liver

Homogenization of the liver samples was accomplished in a buffer solution (10 mM phosphate/20 mM tris-pH 7.0) at 4 °C for 3 min at 600 ×g speed using a mechanical homogenizer. We collected the supernatant to analyze the liver homogenate for oxidant/antioxidant indicators. The colorimetric commercial kits were supplied by the Cairo, Egypt-based Biodiagnostic Co. for this investigation. Following the protocol laid out by Aebi [6], we determined the catalase CAT expression levels. To evaluate superoxide dismutase SOD activity, the methods detailed by Nishikimi et al. [34] were used. Quantitative colorimetric glutathione dehydrogenase (GSH) was performed according to Beutler [13]. The technique created by Uchiyama and Mihara [36] was used to identify malondialdehyde (MDA).

### Evaluation of genes associated with hepatic immunity and cell apoptosis

To extract the total RNA, the frozen liver tissues were processed using the TRIzol reagent (easyREDTM, iNtRON Biotechnology, Korea). The Quantitect^®^ Reverse Transcription kit from Qiagen, Germany, was used to reverse-transcribe the RNA into first-strand cDNA. The sequences of the forward and reverse primers, as well as the housekeeping gene (*β-actin*), are shown in Table 2. In a Rotor-Gene Q thermocycler, we used the following parameters to run the QuantiTect^®^ SYBR^®^ Green PCR kit (Qiagen, Germany): 10 min at 95 °C, followed by 40 cycles of 95 °C for 15 s, 60 °C for 30 s, and 72 °C for 30 s. The purpose of this was to carry out the qPCR analysis. The ^2−ΔΔ^CT method, which was suggested by Livak and Schmittgen [28], was used to ascertain the relative mRNA expression of every gene.

**Table 2:** Oligonucleotide primer sequences

Gene name	Primer sequences		NCBI accession no.	References
***β-actin***	F	TGACCTCACAGACTACCTCATG	XM_003455949.2	**Abarike et al.**, [1]
	R	TGATGTCACGCACGATTTCC		
***TNF-α***	F	CCAGAAGCACTAAAGGCGAAGA	NM_001279533.1	**Standen et al.**, [44]
	R	CCTTGGCTTTGCTGCTGATC		
***IL-1β***	F	TGGTGACTCTCCTGGTCTGA	XM_005457887.3	**Standen et al.**, [44]
	R	GCACAACTTTATCGGCTTCCA		
***TGF- β***	F	GTTTGAACTTCGGCGGTACTG	NM_001311325.1	**Standen et al.**, [44]
	R	TCCTGCTCATAGTCCCAGAGA		
***IL-10***	F	CTGCTAGATCAGTCCGTCGAA	XM_013269189.3	**Standen et al.**, [44]
	R	GCAGAACCGTGTCCAGGTAA		
***Caspase-3***	F	GGCTCTTCGTCTGCTTCTGT	NM_001282894.1	**Standen et al.**, [44]
	R	GGGAAATCGAGGCGGTATCT		
***IFN-γ***	F	GAAACTTCTGCAGGGATTGG	NM_001287402.1	**Velázquez et al.**, [49]
	R	CTCTGGATCTTGATTTCGGG		
***BAX***	F	TACTTTGCATGCCGACTCGT	XM_019357746.2	**Liu et al.**, [26]
	R	CACCTTGCTCCCTGATCCAG		
***BCL2***	F	GACTGTACCAGCCGGACTTC	XM_003437902.5	**Abdelaziz et al.**, [2]
	R	AAAGCAATAATCCGGCCCCA		

Beta actin (*β-actin*, house-keeping gene), interleukin 1beta (*IL-1β*), interleukin 10 (*IL-10*), and interferon gamma (*IFN-γ*), transforming growth factor beta (*TGF- β*), tumor necrosis factor alpha (*TNF-α*), BCL2 associated X protein (*BAX)*, B cell lymphoma-2 *(BCL2)*

### Histopathological investigation

After 48 h of immersion in 10% formalin, the liver, intestines, and spleen samples were dehydrated using ethyl alcohol gradations, washed in xylene, and lastly embedded in paraffin wax to make paraffin blocks. Using a microtome, these blocks were subsequently sliced into 5 μm thickness. Histopathological examination was completed by staining the samples with hematoxylin-eosin [45].

### Test for *Aeromonas sobria* infection

At the end of the 60-day test period, five fish per replication (*N* = 25 fish/group) were exposed to *A. sobria*, a pathogenic bacterium previously found and confirmed in Nile tilapia that had been spontaneously infected in the Department of Aquatic Animal Medicine. Following the manufacturer’s instructions and the descriptions provided by Scheidegger et al. [40]) *A. sobria* was identified at the Department of Microbiology and Immunology, National Research Center (NRC), Dokki, Giza, Egypt, using both traditional biochemical assays and an automated VITEK 2-C15 system for bacterial identification (BioMérieux, France). First documented was the *A. sobria* lethal dosage (LD_50_) according to Almarri et al., [4]. When fish were injected intraperitoneally (IP) with different dosages of live bacteria, the death rate of the infected fish was measured three days later. To elicit 50% fish mortality, the LD_50_ was 2 × 10^8^ CFU/mL. During the bacterial challenge test, a sublethal dosage was administered. The fish were administered a 0.2 mL dose of suspension cells, which contained 1.5 × 10^7^/mL cells, intraperitoneally (IP) using standard MacFarland tubes. To establish accountability for the fish’s demise, *A. sobria* was isolated from the deceased fish. From recently deceased and moribund fish, the bacteria that were injected were re-isolated and identified. For fourteen days, all groups were attentively monitored to keep track of any abnormal findings and daily mortality.

### Analysis of statistical data

Every data set was first tested for normality and treatment-specific variance homogeneity using Shapiro-Wilk and Bartlett’s tests before any statistical analysis was commenced. We compared means using Tukey’s test and performed one-way analysis of variance (ANOVA) for statistical analysis. The level of statistical significance for differences was set at P˂0.05. The statistical package for the social sciences, version 22.0, developed by SPSS Inc. of Chicago, IL, USA, was utilized to analyze the data in the study.

## Results

### Biochemical Findings of VITEK 2-C15 system for *A. sobria* identification

Table 3 illustrates the results of VITEK 2-C15 system for bacterial identification.

**Table 3:** Biochemical picture of *A. Sobria* isolates

Biochemical reaction	Result	Biochemical reaction	Result	Biochemical reaction	Result
Ala-Phe-Pro-Arylamidase	+	β–Glucuronidase	-	Saccharose /Sucrose	-
Adonitol-D-Tagatose	-	β–Xylosidase	-	Sodium Citrate	+
α–Glucosidase	-	5-Keto-D-Gluconate	-	D-Cellobiose	-
β–Galactosidase	+	α–Galactosidase	-	D-Trehalose	+
β-N-Acetyl–Glucosaminidase	+	β-N-Acetyl–Galactosaminidase	-	Malonate	-
β–Glucosidase	-	D-Glucose	+	L-Arabitol	-
β-alanine arylamidasepNA	-	Glutamyl ArylamidasepNA	-	L-Pyrrolydonyl-Arylamidase	-
H2S production	-	Urease	-	Phosphatase	-
Glucose Fermentation	+	γ–Glutamyl–Transferase	-	L-Lactate alkalinization	+
Glycine Arylamidase	-	L-Histidine Assimilation	-	Succinate Alkalinization	+
D-Maltose	+	D-Mannitol	+	D-Mannose	+
Glu-Gly-Arg- Arylamidase	+	Lysine Decarboxylase	+	Ornithine Decarboxylase	-
Courmarate	+	Lipase	+	Palatinose	-
Tyrosine Arylamidase	+	L-Malate Assimilation	-	L-Proline Arylamidase	+
D-Sorbitol	-	L-Lactate Assimilation	-	O/129 Resistance	+
Ellman	+				

### Assessment of growth performance and whole-body composition in fish

The growth performance and feed utilization of *O. niloticus* fed test diets with varying TTO levels are presented in Table 4. At optimal level of 2.0 ml/kg diet, diets supplemented with TTO substantially enhanced growth performance and feed intake in comparison to other groups (*p* < 0.001). In addition, the administration of the diet containing 2.0 ml/kg SEO resulted in a substantially higher final body weight (FBW), weight gain (WG) and specific growth rate (SGR) than the administration of other doses. On the other hand, the FCR of fish that received the 2.0 ml/kg diet was substantially lower than that of fish that received diets containing 0.0, 0.5, and 1.0 ml/kg (*p* < 0.001). The levels of moisture, and ash in the musculature of *O. niloticus* did not undergo any statistically significant changes (*p* > 0.05) as a consequence of the dietary supplementation of SEO for 60 days. The crude protein and crude lipids levels in the muscles of the groups that received a 2.0 SEO/kg diet were significantly changed (*p* < 0.05).

**Table 4:** Effect of Tea Tree oil (TTO) dietary supplementation on growth performance and whole-body composition (% fresh weight basis) of *O. Niloticus* for 60 days

Items	TTO Levels (mL)				*P*-Value		
	Control (0.0)	0.5	1.0	2	Combined	Linear	Quadratic
**Initial body weight (g)**	16.33 ± 0.764	16.57 ± 0.681	15.23 ± 0.681	16.00 ± 1.054	0.275	0.411	0.316
**Final body weight (g)**	44.17 ± 1.069^c^	44.60 ± 0.889^c^	47.83 ± 1.595^b^	59.80 ± 1.513^a^	< 0.001	< 0.001	0.001
**Weight gain (g)**	27.83 ± 0.306^c^	28.03 ± 0.503^c^	32.60 ± 0.917^b^	43.80 ± 0.529^a^	< 0.001	< 0.001	< 0.001
**Specific growth rate (%)**	1.658 ± 0.038^c^	1.651 ± 0.046^c^	1.907 ± 0.019^b^	2.199 ± 0.070^a^	< 0.001	< 0.001	0.057
**Feed intake (g)**	46.33 ± 0.764^c^	46.63 ± 0.513^c^	48.03 ± 0.351^b^	53.50 ± 0.500^a^	< 0.001	< 0.001	0.001
**Feed conversion ratio**	1.664 ± 0.009^a^	1.663 ± 0.024^a^	1.474 ± 0.031^b^	1.221 ± 0.006^c^	< 0.001	< 0.001	0.001
**moisture**	73.42 ± 0.214	73.48 ± 0.161	73.70 ± 0.239	73.36 ± 0.326	0.401	0.806	0.148
**C lipid**	4.637 ± 0.261^a^	4.617 ± 0.202^a^	4.233 ± 0.076^b^	4.130 ± 0.030^b^	0.012	0.003	0.543
**C protein**	16.47 ± 0.126^b^	16.48 ± 0.076^b^	16.69 ± 0.320^b^	17.11 ± 0.091^a^	0.008	0.001	0.332
**ASH**	5.477 ± 0.078	5.417 ± 0.236	5.377 ± 0.216	5.400 ± 0.315	0.955	0.705	0.698

^abcd^Means with different superscript are statistically different. Values are presented asmeans ± SE, and *n* = 8

### Analysis of blood biochemistry

Serum biochemistry profile results are shown in Table 5. The levels of total protein, albumin, and globulin rose significantly (*p* < 0.001) as the TTO level increased (1.0–2.0 ml/kg). On the flip side, triglycerides, glucose, and cholesterol levels were statistically (*p* < 0.001) lower when TTO levels were increased (1.0–2.0 ml/kg) in comparison to other levels. Also, the addition of 2 ml/kg of TTO to the tilapia fish’s feed significantly reduce their serum glucose level (*p* = 0.005). Serum ALT, ALP, AST, and creatinine levels weren’t statistically altered when diet supplemented with different levels of TTO. Diets treated with 2.0 ml TTO/kg showed the lowest levels of serum urea (*p* = 0.020).

**Table 5:** Effect of Tea tree oil (TTO) dietary supplementation on some biochemical indices of *O. niloticus* for 60 days

**Items**	**TTO Levels (mL)**				***P*-Value**		
	**Control (0.0)**	**0.5**	**1.0**	**2**	**Combined**	**Linear **	**Quadratic **
**ALT(U/L)**	23.15±2.663	23.60±1.967	22.75 ± 2.407	22.48 ± 1.381	0.925	0.614	0.904
**AST(U/L)**	26.92±1.535	26.33±0.907	26.98±1.175	26.75±1.146	0.910	0.975	0.910
**ALP(U/L)**	9.500±0.656	9.273±1.074	9.783±0.257	9.033±0.961	0.706	0.571	0.538
**Urea (mg/dl)**	4.100±0.473^a^	4.050±0.328^a^	3.587±0.230^ab^	3.150±0.150^b^	0.020	0.003	0.851
**Creatinine (mg/dl)**	0.437±0.057	0.437±0.051	0.423 ± 0.050	0.353±0.015	0.164	0.043	0.379
**Cortisol (mg/dL) **	17.70±2.007^a^	16.77±0.702^ab^	14.93±0.702^bc^	12.83±0.764^c^	0.005	0.001	0.861
**Glucose (mg/dL) **	42.60±0.800^a^	42.50±1.249^a^	36.10±2.883^b^	33.03±1.332^b^	<0.001	<0.001	0.725
**Cholesterol (mg/dL) **	108.6±1.251^a^	108.7±1.213^a^	100.5±1.803^b^	91.72±1.751^c^	<0.001	<0.001	0.105
**Triglycerides (mg/dL) **	93.60±1.126^a^	92.83±0.764^a^	90.00±1.000^b^	82.17±1.041^c^	<0.001	<0.001	0.007
**T protein (g/dl)**	3.407±0.110^c^	3.420±0.147^c^	4.197±0.125b^b^	4.700±0.200^a^	<0.001	<0.001	0.933
**Albumin (g/dl)**	1.357±0.067^c^	1.303±0.015^c^	1.700±0.100^b^	1.923±0.064^a^	<0.001	<0.001	0.558
**T globulin (g/dl)**	2.050±0.114^c^	2.117±0.159^c^	2.497±0.025^b^	2.777±0.142^a^	<0.001	<0.001	0.818

Values are presented as means ± SE, and *n* = 10

*ALT* Alanine aminotransferase, *AST* Aspartate aminotransferase, *ALP* Alkaline phosphatase

^abcd^Means with different superscript are statistically different

### Immune reactions

**Fig. 1:**
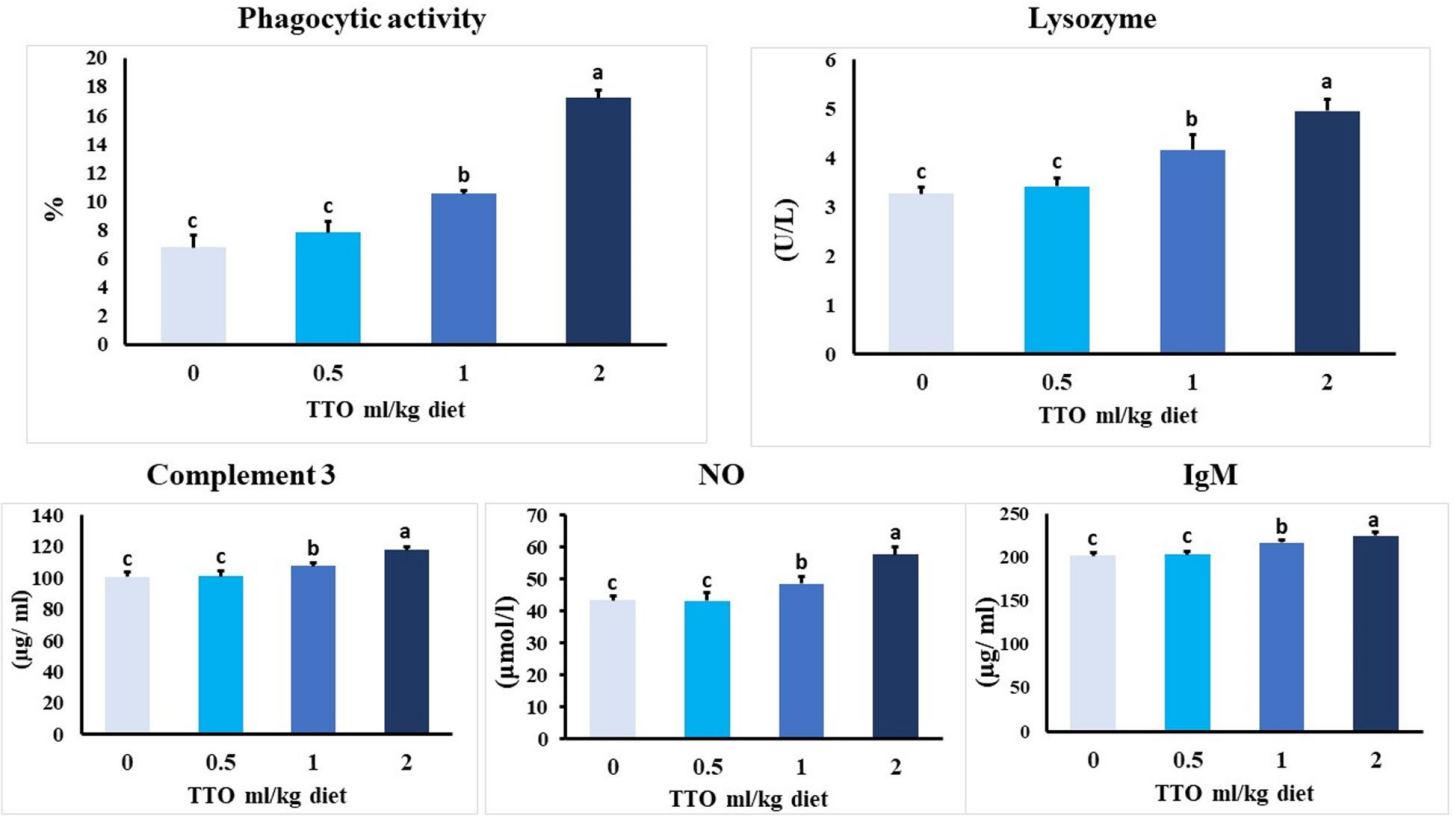
Effect of TTO dietary supplementation on innate immune parameters of *O. niloticus* for 60 days. Data expressed as mean ± SE, *N* = 10 for each group. Columns bearing different letters are significantly different at *p* < 0.001

The dietary TTO had a substantial impact (*p* < 0.001) on the levels of serum lysozyme, NO, C3, and phagocytic activity % in *O. niloticus* fish, as shown in Fig. 1. The inclusion of 2.0 ml TTO/kg food resulted in significantly elevated levels of immune markers compared to all other treated fish groups. No statistically significant differences (*P* > 0.001) were found in the levels of those parameters between the groups that received (0-0.5) ml of TTO per kg of diet.

### Evaluation of hepatic oxidative stress and antioxidant capacity

**Fig. 2:**
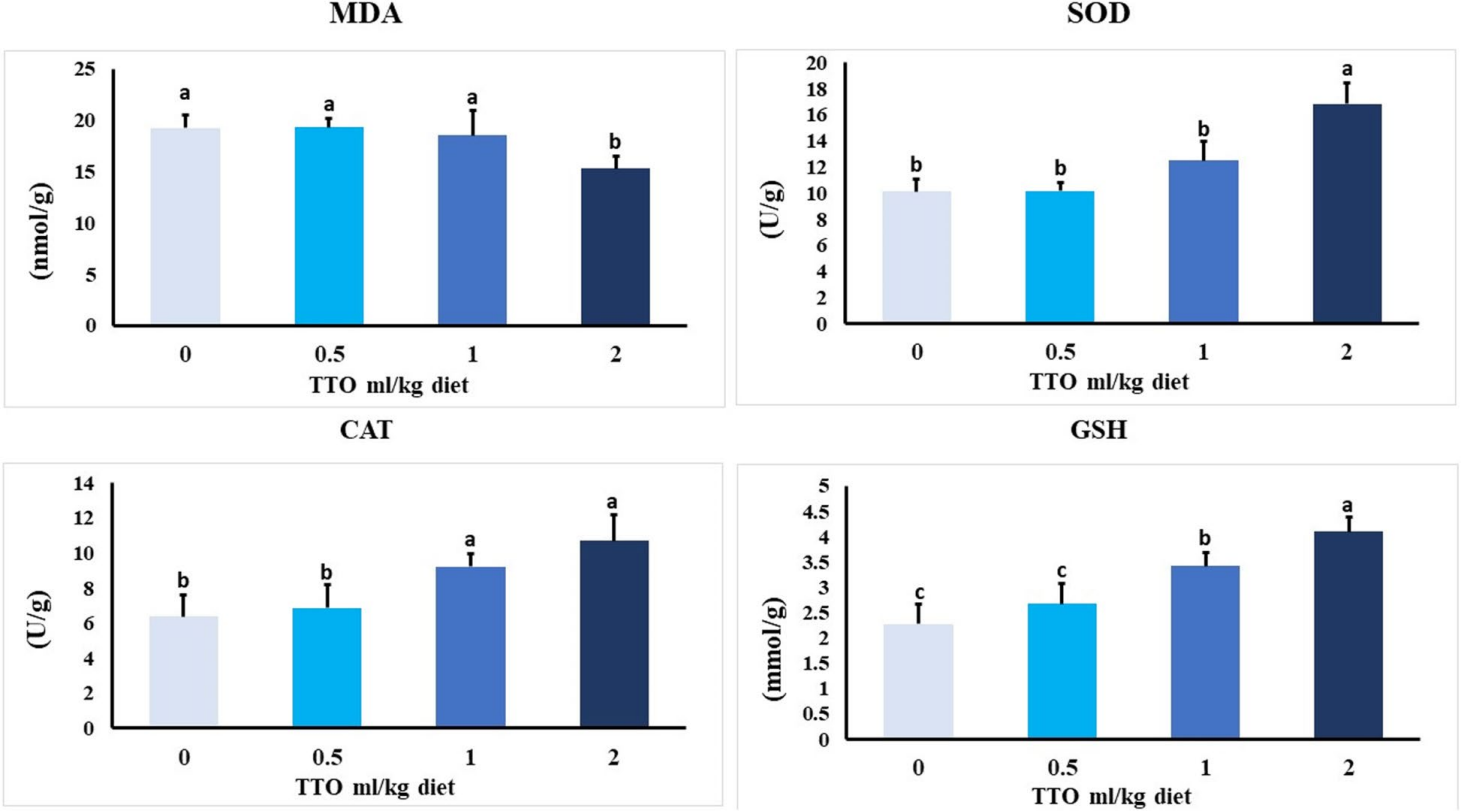
Effect of TTO dietary supplementation on antioxidant biomarkers in the liver tissues of *O. niloticus*. Data expressed as mean ± SE, *N* = 10 for each group. Columns bearing different letters aresignificantly different at *p* < 0.001

The hepatic oxidant/antioxidant enzymes, including superoxide dismutase (SOD), catalase (CAT), glutathione peroxidase (GSH), and malondialdehyde (MDA), were measured in Nile tilapia after a 60-day feeding trial. The results are presented in Fig. 2. According to reports, the activities of SOD, CAT, and GSH increased as the levels of TTO in diets increased. The highest values were seen in the treatment where the diet contained 2.0 ml of TTO per kilogram. In contrast, when the dietary levels of TTO were high, there was a significant drop (*p* = 0.039) in the MDA level. The highest values were observed in the groups that received 0.0–1.0 ml TTO/kg diet.

### Analysis of gene expression patterns

**Fig. 3:**
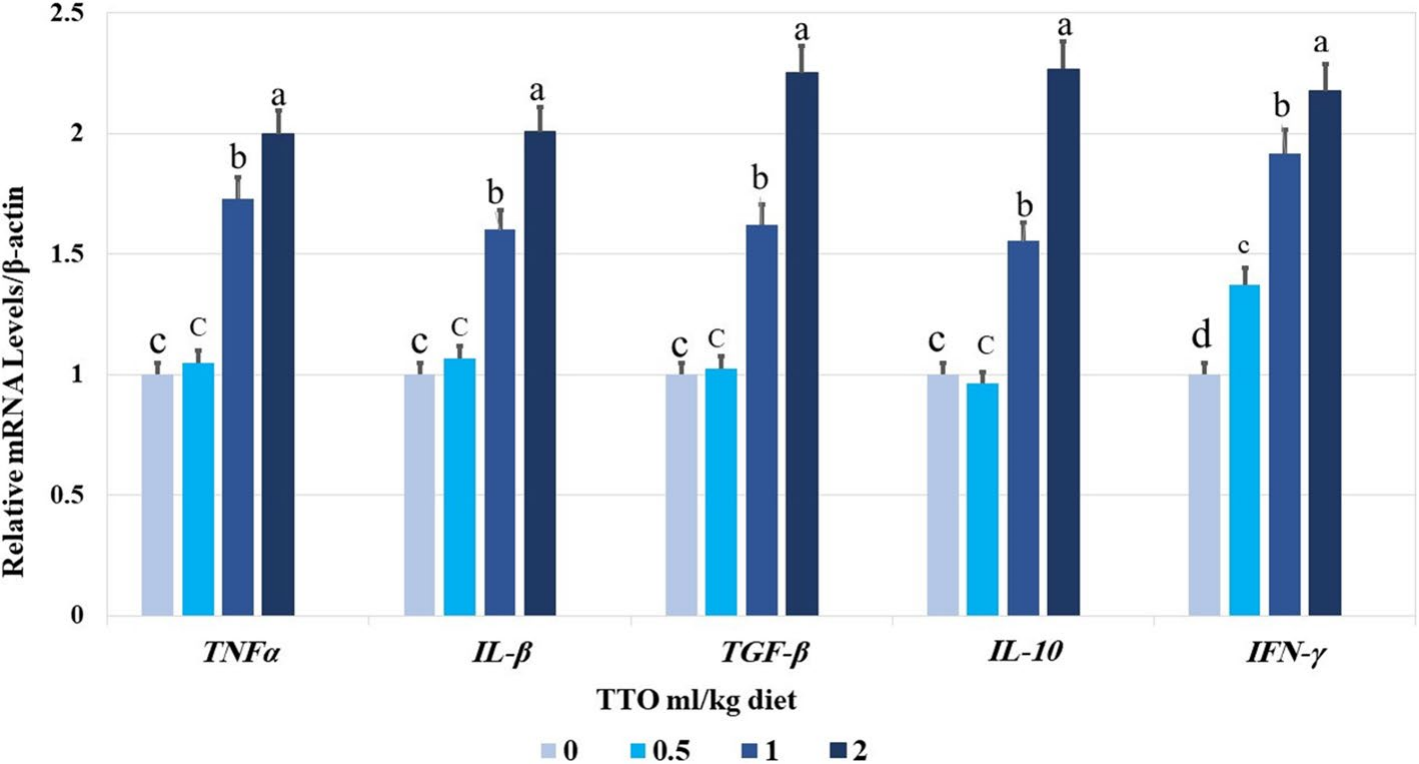
Effect of TTO dietary supplementation for 60 days on mRNA expression of tumor necrosis factor alpha (*tnf-α*), transforming growth factor-beta (*TGF-β*), interferon- gamma (*IFN-γ*), interleukin 10 (*IL-10*), and interleukin 1 beta (*i1- β*) genes in the liver of *Oreochromis niloticus*. Data expressed as mean ± SE, *N* = 10 for each group. Each bar carrying different letters (**a**, **b**, and **c**) was significantly different at *p* < 0.001

**Fig. 4:**
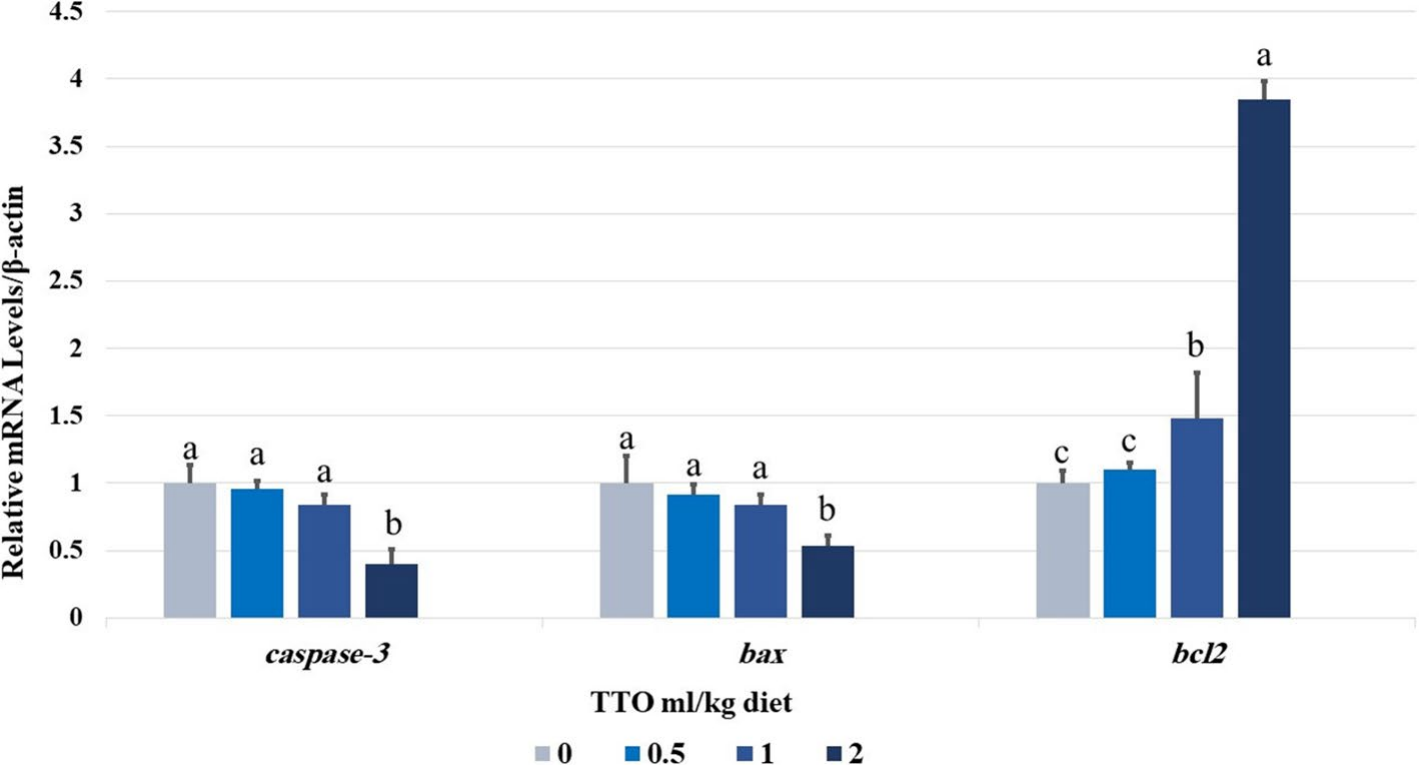
Effect of TTO dietary supplementation for 60 days on mRNA expression of caspase-3, apoptosis regulator *BAX*, *BCL-2*-like protein in the liver of *Oreochromis niloticus*. Data expressed as mean ± SE, *N* = 10 for each group. Each bar carrying different letters (a, b, and c) was significantly different at *p* < 0.001

The genes related to immune-apoptotic response were altered by incorporating TTO supplements for 60 days. Specifically, groups that received 2 ml TTO/kg exhibited significantly higher levels of hepatic *IL-1β*,* IFN-γ*,* TGF-β1*,* IL-10* and *TNF-α* mRNA expression compared to other groups (*p* < 0.001) (Fig. 3). The levels of hepatic *caspase-3* and *BAX* were shown to be lowest when given 2.0 ml TTO/kg diet as dietary supplements (Fig. 4). The groups that received 1&2 ml/kg TTO had significantly higher levels of *bcl2* expression compared to the other groups (*p* < 0.001).

### Histopathological findings

#### Liver

**Fig. 5:**
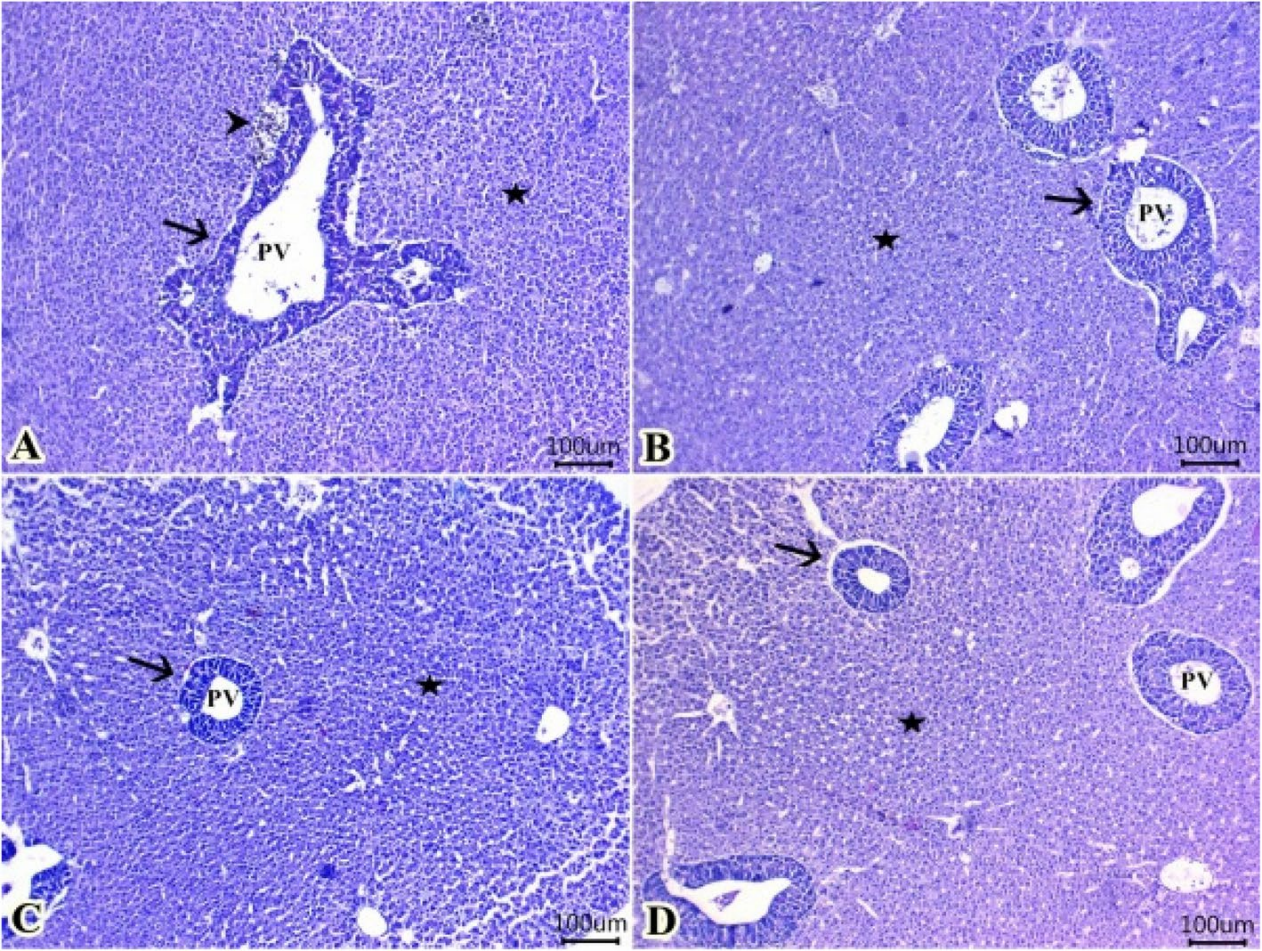
Representative photomicrograph of H&E-stained sections from liver (Scale bar 100 μm) showing: **A** normal histological structures of hepatic cells (stars) around a central vein and groups of pancreatic acini (arrows) around branches of the portal vein (PV) in all groups (**A**, **B**, **C**, **D**). Melanomacrophage centers adjacent to pancreatic acini particularly in control group (arrowhead) (**A**)

Fish groups that fed different levels of TTO showed normal radially arranged hepatic cells around a central vein with narrow hepatic sinusoids. Blood sinusoids were lined by elongated endothelial cells with flattened nuclei. The hepatic cells were mostly oval to polyhedral in shape and vacuolated. Moreover, groups of pancreatic cells arranged in acinar structures around branches of the portal vein. The exocrine cells were columnar epithelium and characterized by spherical basal nuclei with obvious nucleoli and have zymogen granules in the apical ends (Fig. 5A, B, C and D). Melanomacrophage centers may be seen adjacent to pancreatic acini particularly in control group (Fig. 5A).

#### Spleen

**Fig. 6:**
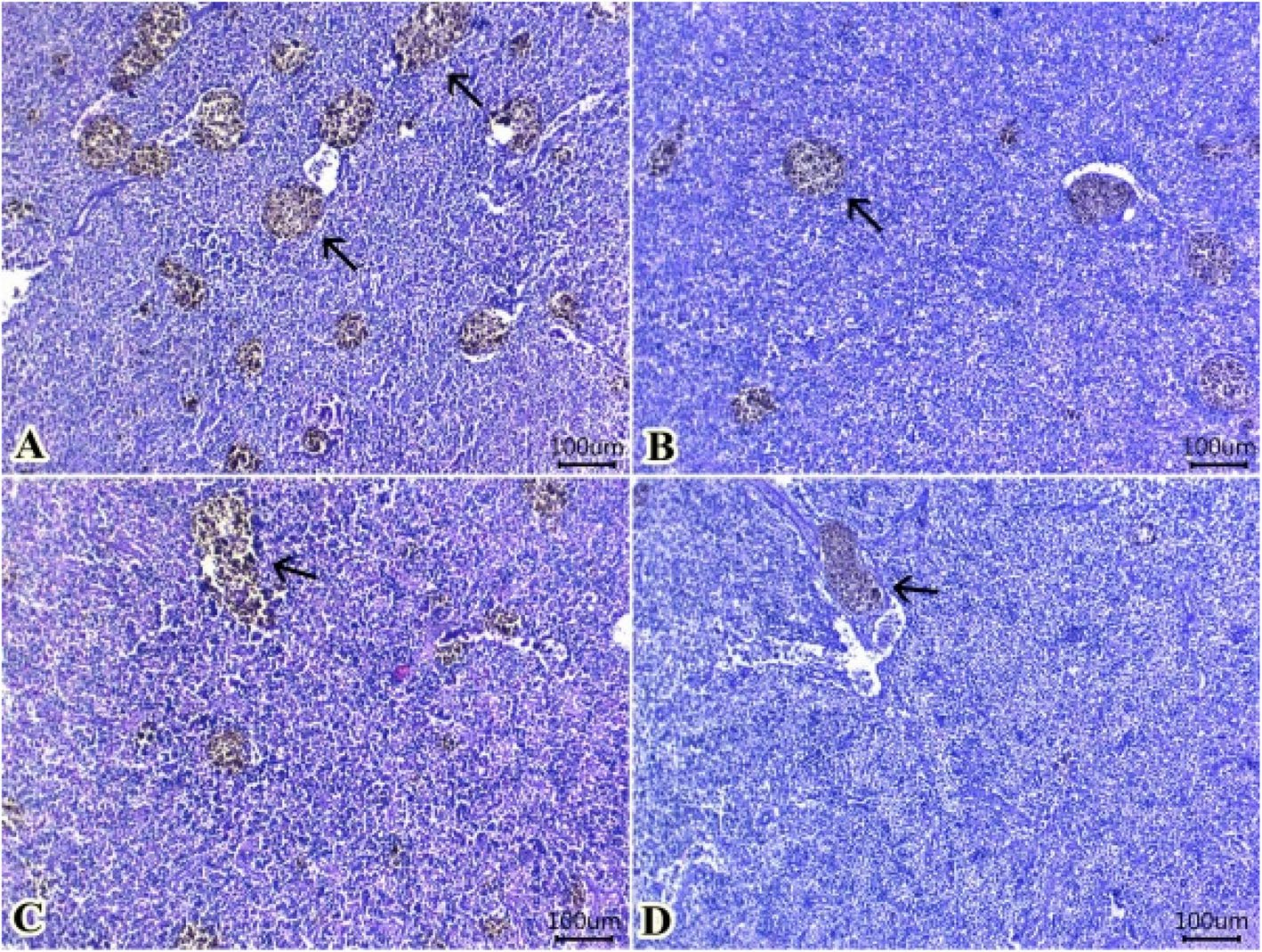
Representative photomicrograph of H&E-stained sections from spleen (Scale bar 100 μm) showing: A: Preserved structures of red pulps, white pulps and sinusoids in all groups (**A**, **B**, **C**, **D**) with numerous foci of melanomacrophage deposits (arrows) around ellipsoids arterioles in group fed 0 ml TTO /kg diet (**A**). Gradually decrease melanomacrophage centers (arrows) at group received 0.5 ml TTO /kg diet (**B**) followed by group received 1.0 ml TTO /kg diet (**C**) then group received 2.0 ml TTO /kg diet (**D**)

Preserved structures of red pulps, white pulps and sinusoids were seen in all groups (Fig. 6A, B, C and D). However, control group that fed 0 ml TTO/Kg diet (Fig. 6A) showed numerous foci of melanomacrophage deposits around ellipsoids arterioles. The melanomacrophage centers were reduced gradually at group fed 0.5 ml TTO/Kg diet (Fig. 6B) followed by group fed 1.0 ml TTO/Kg diet (Fig. 6C) and group fed 2.0 ml TTO/Kg diet (Fig. 6D).

#### Intestine

**Fig. 7:**
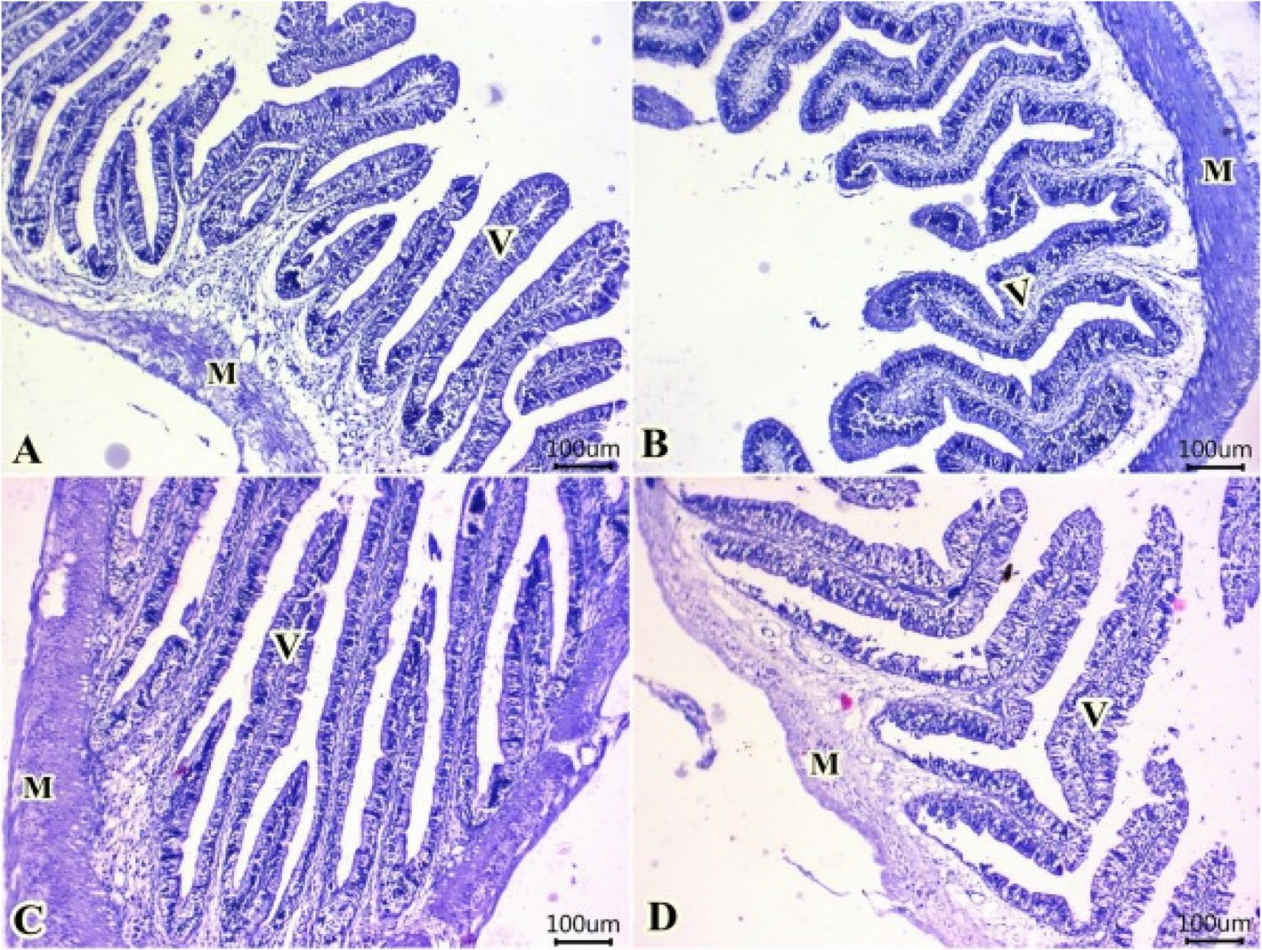
Representative photomicrograph of H&E-stained sections from intestine (Scale bar 100 μm) showing: normal configurations of simple columnar enterocytes lining mucosal villi (V), submucosal layer and muscular layer (M) in all groups (**A**, **B**, **C**, **D**)

**Fig. 8:**
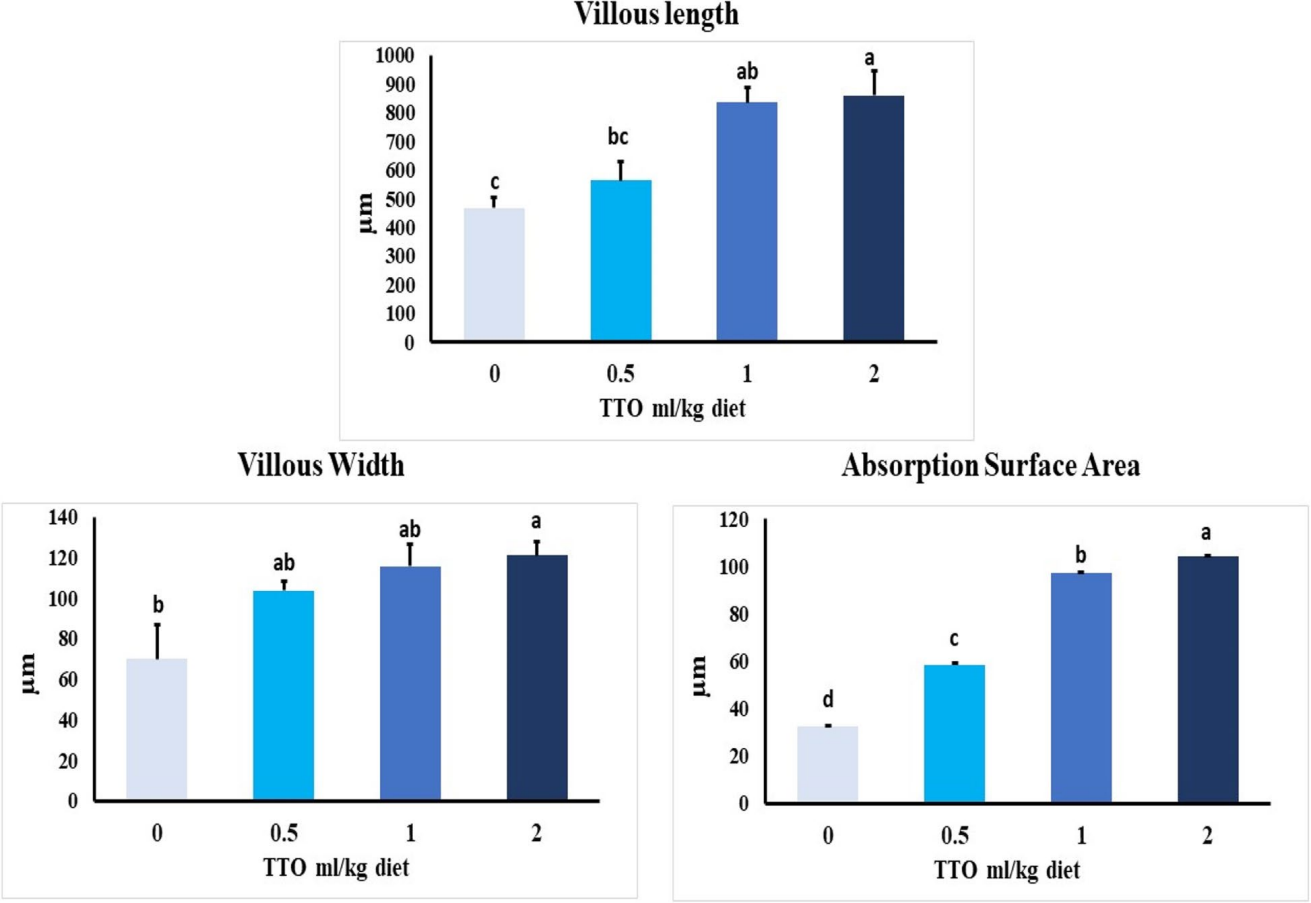
Intestine morphometry of *O. niloticus*, fed with diets enriched with different levels of TTO for 60 days. Bars assigned by different letters are significantly different at *P* < 0.001

All groups demonstrated normal structure of simple columnar enterocytes lining mucosal villi, submucosal layer and muscular layer (Fig. 7). Furthermore, enhancement in histomorphological structures of intestinal layers particularly villi were observed in all treated groups (Fig. 8). At which, values of intestinal parameters as villus length (VL), villus width (VW), absorption surface area (ASA) were increased gradually at group fed 0.5 ml TTO/Kg diet followed by group fed 1.0 ml TTO/Kg diet. High levels of intestinal parameters were seen in group fed 2.0 ml TTO/Kg diet (Fig. 8).

### Bacterial challenge

**Fig. 9:**
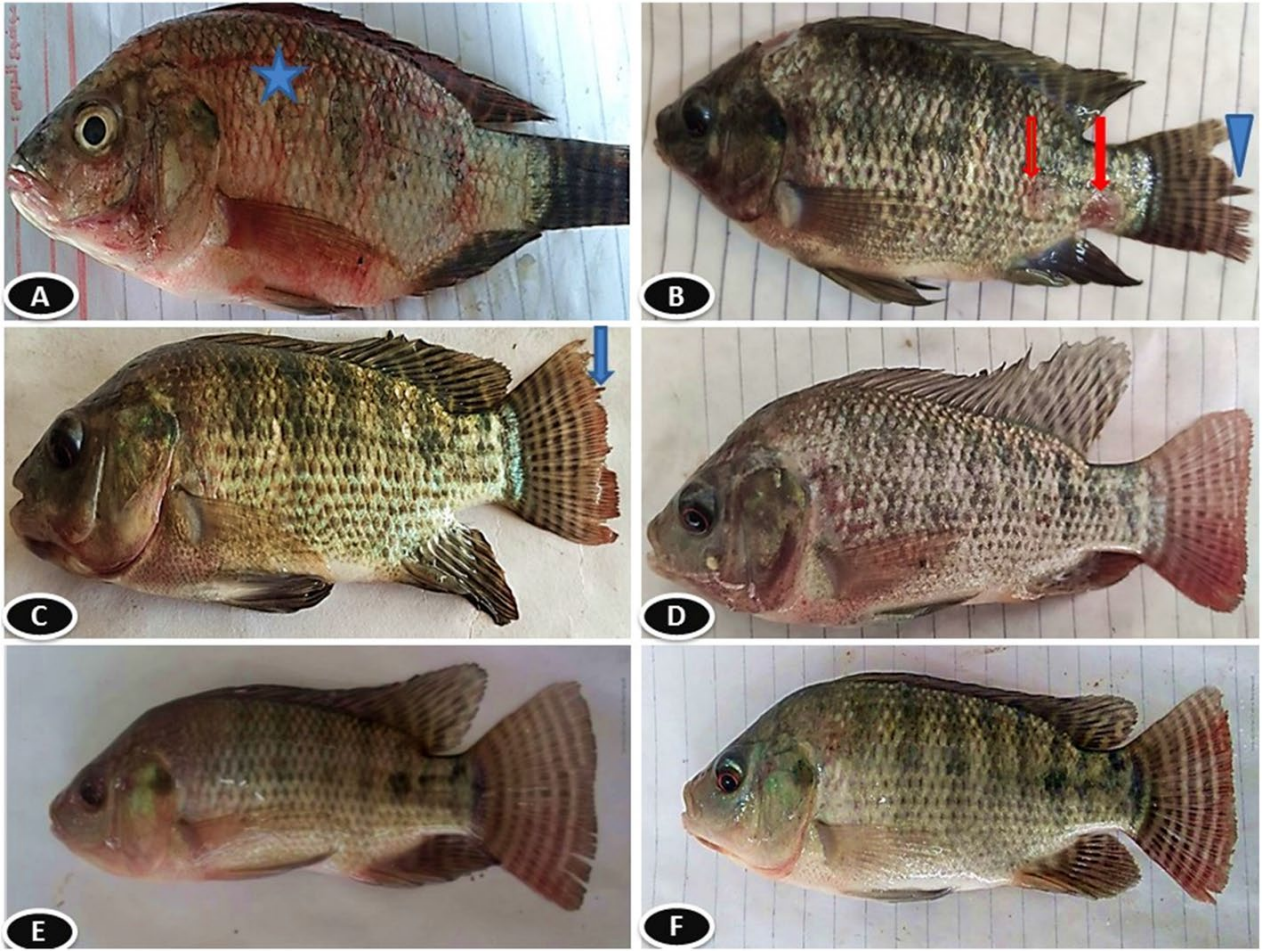
Clinical observation of *O. niloticus* after 14 days of *A. sobria* challenge, following a 60-day diet supplemented with TTO showing: control fish which received 0 ml TTo/Kg exhibited sever body hemorrhages (star) (**A**) and fin rot (arrow head), and skin ulcers (red arrows) (**B**). Fish received 0.5 ml TTo/kg diet exhibited mild fin rot (blue arrow) (**C**). Fish received 1.0 ml TTo/kg diet exhibited mild fin hemorrhage (**D**). Fish received 2.0 ml TTo/kg diet exhibited normal appearance (**E**) and (**F**)

Table 6 shows that after 60 days of feeding Nile tilapia diets supplemented with TTO, their resistance to the *Aeromonas Sobria* challenge was determined by evaluating mortality %, RPS, and clinical and post-mortem symptoms. The mortality rate was highest in the control group at 72% and lowest in the group that received 2.0 ml TTO/kg diet at 12%. There was also a dose-dependent increase in RPS when TTO was added to Nile tilapia’s diet. The group that received 2.0 ml SEO/kg diet had the greatest RPS (83.33%). Table 5 illustrates that control groups exhibited reduced appetite, anomalous swimming behavior, and reflex impairment, as well as post-mortem alterations in *O. niloticus* challenged fish. Conversely, other TTO-supplemented groups at dose of 1&2 ml/Kg experienced a gradual decline in these characteristics. Figure 9 shows clinical observation of *O. niloticus* after 14 days of *A. sobria* challenge, following a 60-day diet supplemented with TTO.

**Table 6:** Relative percent survival, clinical signs and post-mortem findings observed in survived O. Niloticus fish in different experimental groups challenged with Aeromonas Sobria

Tea tree oil (TTO) levels ml/kg diet					
		**0**	**0.5**	**1**	**2**
**No. of survived fish**	Number	7	11	17	22
**Survival rate**	%	28	44	68	88
**Mortality rate**	%	72	56	32	12
**Relative percent survival**	%	0	22.22	55.55	83.33
**Abnormal swimming activity**	Number	5/7	6/11	4/17	3/22
	Score	+++	++	+	+
**Loss of appetite**	Number	5/7	5/11	3/17	0/22
	Score	+++	++	+	-
**Loss of reflexes**	Number	4/7	5/11	3/17	2/22
	Score	++	++	+	+
**External skin lesion**	Number	6/7	5/11	4/17	2/22
	Score	++++	++	+	+
**Postmortem change**	Number	5/7	4/11	3/17	0/22
	Score	+++	++	+	-

The score of symptoms were recorded as follows: (-) no; (+‏) weak; (‏‏++) mild; (+++) moderate; (++++‏‏‏) severe.

## Discussion

The Myrtaceae family is one of the crucial essential oils-enriched plant families, and *Melaleuca* is a famous genus of this family with enriched EOs. The essential oils which are extracted from the major species of this genus such as *M. quinquenervia*,* M. alternifolia*,* M. cajuputi*, and *M. bracteata*, are subjected to oil extraction, is named tea tree oil (TTO) worldwide [35]. The application of TTO has expanded in different industries such as fungicide, preservative, in cosmetics, aromatherapy, and herbal medicines [52]. Tea tree essential oil is being recently consumed in the aquaculture field as growth promoter and for boosting health and performance of fishes [16, 27]. Additionally, it is successfully tested as a natural antimicrobial agent against *Aeromonas* spp. (Mumu and Hossain, 2018). Therefore, the goal of the current study was to value various doses supplementation on growth performance, protein/lipid profile, stress condition, immune function, antioxidant capacity, hepato-renal function, histopathological picture, gene function of Nile tilapia, and bacterial resistance.

In the present study, we detected enhanced growth performance with a TTO supplementation in a dose-dependent manner. Current research by Liu et al. [25] supported our findings and reported that dietary TTO induced better growth performance in *Macrobrachium rosenbergii* because of its role in enhancing lipid metabolism. Aydin et al. [9] added that the basic role of lipid metabolism is to deliver energy for growth and organs’ development. Earlier studies supported our findings and reported that TTO is a beneficial feed additive which can boost growth performance, feed conversion ratio, and innate immunity [7, 10]. The growth promoting activity of TTO could be returned to its mechanism of action which is represented in efficiently improving the structure of intestinal flora, suppressing the growing of harmful bacteria, and enriching the colonization capacity of useful bacteria in the intestine [15].

Measurements of total serum protein, lipid, and stress indicators are crucial for reflecting fish health condition and immune function. Monitoring total protein content in fish serum is a reflection for the humoral immune response to bacterial diseases (Maqsood et al. [33]). Also, augmenting levels of blood protein indicator an improved survival rates and immunological function in fish (Simanjuntak et al. 2018). Herein, it was noted a supreme value of total protein, globulin, and albumin as well as declined levels of lipids (cholesterol and triglycerides), and stress indicator (cortisol) distinguished in the supplementing group by TTO at 2.0-mL per kg diet reflecting enhanced immune response. This modulating effect could be attributed to the major role of TTO in regulating protein and lipid metabolism and accordingly, lessening the inflammatory response and stress condition as reported by Yang et al. [53].

Investigating hepatic and renal functions is crucial to clarify the role of plant extracts and their essential oils [11]. In the present work, supplementing TTO especially at 2.0-mL per kg diet has a hepato-renal protecting function indicated by declining activities of ALT, AST, ALP, urea, and creatinine levels. Such improvement in the renal and liver function could be returned to the antioxidant activity triggered by the components of TTO. A recent study by Liu et al. [24] elucidated that tea tree oil can alleviate oxidative damage via triggering the NF-κB/NO pathway. Concurrently, Liu et al. [26] verified the hepato-protecting effect of TTO which was indicated by modulating levels of ALT and AST in TTO-subjected groups in largemouth bass.

Assessment of immune function has a potential role for addressing the efficacy of natural herbals as well as to record the tolerance of fish after bacterial challenge [31]. In the current study, we reveal augmenting values of immune biomarkers including phagocytic activity, complement, lysozyme, No, and IgM Additionally, the resistance against *A. sobria* was highly noticed in a dose-dependent manner with the maximum result at highest applied dose (2.0 mL per kg diet). Such dose proved the best outcome in elevating survivability percentage and improving clinical signs and post-mortem findings. In line with an early report, Souza et al. [42] supported our findings and reported that TTO developed the immune response in silver catfish following infection by *A. hydrophila*.

Considering oxidative stress, antioxidant response, and hepato-renal functions are essential to assess the possible role of herbal extracts on fish’s health condition and against bacterial infection [32]. Outcomes exhibited that supplementing TTO in fish diet had a promising role in modulating levels of antioxidants (CAT, SOD, and GSH) levels especially in the group given TTO at 2.0-mL per kg diet after 60 days reflecting potent antioxidant activity. In addition, dietary interfere by TTO diminished value of oxidant indicator (MDA). Such outcome was in harmony with Liu et al., [26] who detected an elevation in CAT activity and a reduction in MDA upon supplementing largemouth bass dietary 1 g /kg of TTO. The potential effect of tea tree oil could be dominated to its antioxidant constituents of α-terpinene and terpinen-4-ol. Theses constituents can directly boost antioxidant competences via lessening levels of decrease reactive oxygen species and free radical in various tissues as early clarified by Rudbäck et al. [38] and Souza et al. [43].

Assessment of gene function plays a mandatory role for the assessment of natural herbals and their extracted essential oils [20]. In the current study, dietary inclusion of TTO has verified immune-regulatory and anti-inflammatory roles which were shown in elevating values of *TNF-α*,* IL- 1β*,* TGF- β*,* IFN-γ* and *IL-10* genes in a dose-dependent manner. Likely, Yang et al. [53] found enhanced TNF-α and IL-6 upon TTO treatment indicating anti-inflammatory effect. Also, it has been detected that TTO has an essential role in preventing apoptosis of CASP3 and protecting against cytotoxicity which was indicated by inhibiting the expression of CASP3. Additionally, TTO-supplementation especially at a higher dose revealed a weak caspase-3 and strong BCL2 gene expression indicating the crucial role of TTO in maintaining tissue homeostasis as reported by Raducka-Jaszul et al. [37]. On the same manner, Liu et al. [25] found improved the expression of antioxidant genes following supplementing *Macrobrachium rosenbergii* by TTO implying to the antioxidant protecting role of TTO.

Histological assessment can be used as one of the parameters to reflect the effectiveness of the antioxidant property of the natural plant extracts [19]. The present data demonstrated that the highest concentration of TTO (2.0-mL /kg diet) showed great values of histomorphological parameters in intestine including villus length, villus width, and absorption surface area. This could be attributed to the active antioxidant ingredients of tea tree oil (terpenes, mainly monoterpenes) as reported by Carson et al. [14]. Assisting this explanation, Baldissera et al. [12] reported that TTO regenerated histo-pathological injury in gills following infection of silver catfish by *A. hydrophila.*

## Conclusion

In conclusion, supplementing TTO in fish diet at 2.0 mL/kg reflects a favorable influence on improving growth efficacy, biochemical patterns including antioxidant capacity, hepato-renal function, stress state, lipid and protein profiles, gene expression, and histological structure. Hence, it is endorsed to utilize TTO supplementation for sustaining health and performance of Nile tilapia particularly for protecting fish against bacterial infection. Further reports are required to assess other effects of TTO on other fish species.

## Data Availability

All data are available in this manuscript.
